# Development of a master of science in digital health program at Muhimbili University of Health and Allied Sciences, Tanzania: a mixed-methods curriculum development study

**DOI:** 10.3389/fpubh.2026.1895664

**Published:** 2026-10-05

**Authors:** Felix Sukums, Linda Simon Paulo, Yoktan Thadeo, Mpanga Paul, Samwel Mndima, Juliana Mbuke, Rebecca Chaula, Junior Kaskazi, Amani Anaeli, Obadia Nyongole

**Affiliations:** Muhimbili University of Health and Allied Sciences, Dar es Salaam, Tanzania

**Keywords:** artificial intelligence, curriculum development, digital health, Tanzania, workforce

## Abstract

**Background:**

The rapid expansion of digital technologies is transforming health systems and creating increasing demand for a workforce with competencies in health informatics, data science, artificial intelligence (AI), cybersecurity, digital health governance, and implementation. However, digital health training remains fragmented in many low- and middle-income countries (LMICs), including Tanzania. This study describes the systematic, evidence-based process used to develop an MSc Digital Health program at Muhimbili University of Health and Allied Sciences (MUHAS), Tanzania.

**Methods:**

A mixed-methods curriculum development approach was undertaken, comprising document review and benchmarking, a quantitative stakeholder survey, qualitative interviews with alumni of the MSc Health Information Management program, and a stakeholder consultative workshop. Nine higher-education and competency-framework sources were reviewed to identify gaps and emerging competency domains. A structured survey was administered to 48 stakeholders purposively selected from government, non-governmental, private-sector, and start-up organizations. Qualitative data from alumni interviews and open-ended survey responses were analyzed thematically using manual coding by two independent coders. A two-day consultative workshop involving 22 stakeholders from academia, professional associations, non-governmental organizations, government, and clinical practice reviewed and validated the proposed curriculum.

**Results:**

Benchmarking demonstrated substantial variability in digital health curriculum content, with no single program comprehensively covering all priority competency domains. Stakeholders identified AI and machine learning (48.0%) and big data and analytics (41.7%) as prominent emerging technology competencies, while data science and predictive analytics, health information systems and interoperability, cybersecurity and data privacy, and epidemiology and biostatistics were also emphasized. Overall, 81.3% of survey respondents considered digital health competencies highly important to their organizations, while 89.6% indicated willingness to employ MUHAS MSc Digital Health graduates. Qualitative findings emphasized five priority areas: advanced computing and emerging technologies; applied health data science and analytics; health systems architecture and interoperability; experiential learning and program delivery; and governance, cybersecurity, leadership, and professional ethics. Workshop participants endorsed the proposed curriculum and recommended stronger practical learning, field attachment, innovation-oriented projects, and alignment of course content with emerging workforce needs. The resulting program was structured as a two-year, full-time MSc Digital Health program with three specialization tracks: Digital Health Leadership, Digital Health Systems Development, and Health Data Science and AI.

**Conclusion:**

The MUHAS MSc Digital Health curriculum was developed through an iterative, evidence-informed, and stakeholder-driven process that integrates multidisciplinary digital health competencies with practical, implementation-oriented learning. The approach provides a potentially adaptable model for curriculum development in LMIC settings. Future evaluation should assess program implementation, graduate competencies, employment outcomes, employer satisfaction, and contribution to national digital health transformation.

## 1 Introduction

The advancement of digital technologies has transformed health systems globally, offering new opportunities to improve efficiency, quality of care, and population health outcomes. The WHO emphasizes the integration of digital technologies as a critical enabler for strengthening health systems and achieving universal health coverage (UHC) and the Sustainable Development Goals (SDGs), particularly SDG 3 (1, 2). However, the successful implementation of digital health interventions depends on the availability of a skilled and adequately trained workforce.

Despite growing investments in digital health, low- and middle-income countries (LMICs), particularly in Africa, continue to face substantial workforce shortages. LMICs are estimated to face a shortfall of approximately 10 million healthcare workers by 2030, with Africa alone requiring an additional 6.1 million workers (1). Beyond general workforce shortages, a significant gap remains in digital health competencies, including data analytics, health information systems, and emerging technologies such as AI (3). Global assessments, including the Global Digital Health Index (GDHI), consistently identify workforce capacity as one of the weakest domains in digital health readiness (4).

In 2025, the WHO Science Council recommended developing a global digital competency framework in collaboration with Member States and academic institutions. The report also recommended that global, regional and national accrediting bodies and academic institutions review health and allied pre- and in-service curricula to include digital health and relevant technology skills. It further recommended that Ministries responsible for Health, Education, and Finance, as well as professional associations, recognize certificates and offer incentives, and that WHO, in collaboration with Member States, support the development of career paths for digital health professionals to maximize the use of digital technology and bridge workforce shortages.

Similar recommendations from African digital health literature highlight the importance of coordinated collaboration among ministries responsible for health, education, and ICT, alongside academic institutions and professional bodies, to formalize digital health curricula and strengthen workforce preparedness (5, 6).

In Tanzania, these challenges are reflected in limited digital literacy among healthcare professionals and insufficient integration of digital competencies into training programs. National frameworks such as the Tanzania Development Vision 2025 and the Tanzania Digital Health Transformation Strategy (2025–2030) emphasize the role of information and communication technologies in strengthening health systems and improving service delivery (7, 37, 38). Despite these efforts, gaps in digital health workforce capacity remain a major constraint to effective implementation.

Muhimbili University of Health and Allied Sciences (MUHAS), as a leading health sciences institution in Tanzania, plays a central role in training the health workforce and conducting research. Through collaborations with national and international partners, MUHAS has increasingly engaged in digital health capacity building and innovation initiatives. Multi-stakeholder partnerships involving government institutions, academia, and development partners have further supported national digital health transformation efforts through knowledge exchange, capacity development, and innovation platforms.

In response to these needs, MUHAS initiated the transition from the MSc Health Information Management program to a more comprehensive MSc Digital Health program. This transition reflects a shift from traditional health records management to a multidisciplinary digital health ecosystem that incorporates data science, AI, and clinical informatics. The initiative draws on the GDHI framework, particularly Indicator 9 (integration of digital health into pre-service training), Indicator 11 (availability of trained professionals), and Indicator 12 (establishment of career pathways) (4); a formal quantitative mapping of the curriculum against GDHI maturity levels was not undertaken and is identified as an area for future work (see Section 5).

This study describes the systematic, evidence-based process used to develop an MSc program in Digital Health at MUHAS, Tanzania, guided by empirical evidence, stakeholder engagement, and alignment with national and global priorities. Consistent with this scope, the manuscript reports on the curriculum development process itself; it does not report program implementation or workforce outcome data, which will be the subject of future evaluation.

## 2 Methods

A mixed-methods approach, comprising document review and benchmarking, a quantitative survey, qualitative key-informant interviews, and a stakeholder consultative workshop, was used to ensure that the revised curriculum was evidence-based and responsive to workforce needs. The components are described below in the order in which they were conducted and are reported in Section 3.

### 2.1 Document review and benchmarking of existing curricula

A comprehensive gap analysis of the existing MSc Health Information Management curriculum was conducted by comparing its content against international academic programs, WHO guidance, and national policy frameworks. Nine comparator higher-education programs in digital health and health informatics were identified and reviewed (Table 1). Programs were selected using the following criteria: regional relevance to East Africa, international recognition/accreditation, comparability of qualification level (MSc), and publicly available curriculum documentation. The rationale for including specific international programs was representation of both LMIC and high-income settings, or prior institutional collaboration. We retrieved curriculum documents, publicly available program handbooks, and module descriptions through institutional websites, direct correspondence with program coordinators, and/or database searches. Four researchers extracted competency domains and topics using a structured extraction template and grouped them into thematic categories through iterative comparison and consensus discussion.

**Table 1:** Summary of topic representation by region/institution.

Region/Institution	Frequency (n)	Percentage (%)
South Africa – Data Analytics	17	6.39%
MSc Health Information System	19	7.14%
East Africa (IUCEA)	88	33.08%
Faculty European Campus Rottal-Inn	75	28.2%
Bournemouth University	27	10.15%
Iran (PhD in HIM competence domains)	37	13.91%
Other institutions	3	1.13%

### 2.2 Quantitative stakeholder survey

A structured quantitative survey was administered to stakeholders from government institutions, non-governmental organizations, private-sector organizations, and start-up organizations to assess workforce needs, required competencies, and perceptions of the relevance of digital health training. The questionnaire was researcher-developed and informed by established digital health competency frameworks, including the World Health Organisation (WHO) digital health competency framework and the Health Information Technology Competencies (HITCOMP) framework, with additional domains informed by the International Medical Informatics Association (IMIA) recommendations (33, 36, 40). These frameworks provide relevant competency domains for digital health workforce education, including informatics and information management, data and analytics, technical skills, communication, ethics and privacy, health information systems, leadership, and digital health technologies.

The questionnaire comprised 20 questions/items organized into sections covering stakeholder and organizational characteristics, perceptions of digital health skills, assessment of specific digital health competencies, emerging digital health technologies and trends, and internship and employment opportunities for MSc Digital Health graduates. Competency items were assessed using a 5-point Likert scale ranging from 1 (Not Important) to 5 (Very Important). The questionnaire assessed the perceived importance of competencies including data analytics, electronic health records, telehealth, software development, data protection and privacy, monitoring and evaluation, health information systems, health technology assessment, legal and ethical issues, communication and interpersonal skills, problem-solving and critical thinking, evidence-based decision-making, project management, leadership, innovation and entrepreneurship, artificial intelligence and machine learning, blockchain, Internet of Things, and wearable technologies. Open-ended questions were included to capture stakeholders’ views regarding changing digital health skill requirements, emerging technologies and additional competencies that should be incorporated into the MSc Digital Health curriculum.

The questionnaire also assessed stakeholders’ willingness to accept MSc Digital Health graduates from MUHAS for internships, employment, or consultancy opportunities. The survey was administered electronically using Google Forms and was contextualized to the Tanzanian digital health workforce and the objectives of the MSc Digital Health curriculum review.

Respondents were identified and recruited through purposive sampling of institutions known to employ health information/digital health professionals, followed by snowball referral. Eligible respondents were individuals with direct experience of health information systems, digital health implementation, or supervision of health informatics graduates.

### 2.3 Qualitative key-informant interviews with alumni

Key informant interviews were conducted with alumni of the MSc Health Information Management program as part of a university-wide tracer study to explore perceived gaps in the previous curriculum, workplace challenges, and recommendations for improvement (8). A total of eight (8) alumni were interviewed. Alumni were identified and selected through purposive sampling stratified by graduation year and sector of employment. Interviews followed a semi-structured guide covering curriculum relevance, workplace application of skills, and suggestions for revision. Interviews were conducted *in person and by telephone*, audio-recorded with participant consent, and transcribed verbatim prior to analysis.

### 2.4 Stakeholder consultative workshop

A consultative workshop was convened on 6th – 7th Nov 2023 to review and validate the proposed MSc in Digital Health curriculum structure, competency domains, learning outcomes, and duration, in accordance with Tanzania Commission for Universities (TCU) guidelines for curriculum development and accreditation. Twenty-two participants were purposively invited to represent academia, industry, and government institutions, with the following institutional representation: the majority were from academic institutions (*n* = 13, 59.1%), followed by professional associations and non-governmental development organizations (*n* = 5, 22.7%), government ministries and public sectors, and clinical practice (*n* = 4, 18.1%).

The workshop involved a plenary discussion followed by small-group discussions with domain experts. Consensus on proposed revisions was reached through discussion and consensus (nominal group technique). Table 2 summarizes the curriculum modifications made as a direct result of the workshop.

**Table 2:** Key outcomes of stakeholder workshop validation.

Curriculum element	Stakeholder feedback	Final decision/action
Public health foundation	Suggested reducing the number of public health courses	Foundational public health courses were retained to provide essential competencies, particularly for students from diverse academic backgrounds
Innovation and dissertation	Recommended greater emphasis on innovative projects and a project-oriented dissertation	Innovative projects were integrated into specialization courses, with feasible projects carried forward into the dissertation.
Practical training	Requested additional practical and innovation-focused learning	Practical hours and learning activities were strengthened, including case-based and hands-on learning
Field-based learning	Emphasized the need for real-world practical exposure	A mandatory digital health systems and technologies field attachment was incorporated
Course and module naming	Suggested reviewing course and module names to ensure alignment with their content	Course names were reviewed and retained as they adequately reflected the digital health focus.
Overall curriculum scope	Emphasized the need for a curriculum responsive to emerging technological developments and workforce needs	The program was finalized as a two-year, full-time MSc Digital Health program, with expanded content and a 54.6% increase in total credits.

### 2.5 Data analysis

Quantitative survey data were analyzed using descriptive statistics (frequencies and percentages) in the Statistical Package for the Social Sciences (SPSS) version 27. Qualitative data from alumni interviews and open-ended survey responses were analyzed using thematic analysis. Two independent coders coded transcripts and responses manually; codes were grouped into candidate themes, then reviewed and refined through discussion between coders and cross-checked against the original data to ensure they reflected participants’ accounts. Disagreements were resolved through discussion and consensus.

### 2.6 Ethical considerations

Ethical clearance for the university-wide tracer study was obtained from the Research and Ethics Committee of Muhimbili University of Health and Allied Sciences (MUHAS), reference number MUHAS-REC-10-2022-1392. Informed consent was obtained from all survey, interview, and workshop participants prior to data collection; participants were informed of the study purpose, their right to withdraw at any time without penalty, and how their data would be used and stored. Participation was voluntary throughout. Illustrative quotations presented in Section 3.3 are anonymized and are not attributable to identifiable individuals; no other personally identifying data are reported in this manuscript.

## 3 Results

### 3.1 Benchmarking of global digital health programs

Content related to digital health was reviewed across nine higher-education and allied institutions. The largest share of identified topics (33.08%) came from the Inter-University Council for East Africa (IUCEA) Benchmarks for the Master of Science in Health Informatics Programs in East Africa, followed by the Faculty of the European Campus Rottal-Inn (28.2%) and a PhD in Health Information Management competence-domain framework from Iran (13.91%); other institutions contributed smaller shares (Table 1).

The benchmarking exercise, comparing AI, data science, and programming competencies across the reviewed programs, revealed substantial variability in curriculum coverage; no single institution demonstrated comprehensive inclusion of all key competency domains. Programs in East Africa primarily emphasized foundational areas, including basic AI concepts, healthcare applications, and introductory programming. In contrast, the Faculty of the European Campus Rottal-Inn demonstrated stronger coverage of advanced competencies, including evaluation of AI algorithms, supervised and unsupervised machine-learning techniques, software architecture, and health information system design. Other programs showed a more specialized focus: South African data-analytics programs concentrated largely on core data-mining concepts. In contrast, other frameworks uniquely incorporated the practical implementation of machine-learning models. The previous MSc Health Information Management curriculum at MUHAS, like several other programs reviewed, showed limited coverage of advanced domains such as AI, machine learning, and applied data science. Overall, the benchmarking findings indicate a fragmented and uneven distribution of competencies across institutions, with no harmonized standard for integrating emerging digital health skills; this fragmentation directly informed the domains prioritized in the revised MUHAS curriculum (Section 3.5).

### 3.2 Quantitative survey findings

Of the 48 survey respondents (age range 22–70 years), 34 were male (70.8%), and 14 were female (29.2%). Participants represented academic institutions (*n* = 17, 35.4%), government ministries and public healthcare facilities (*n* = 14, 29.2%), non-governmental and international development organizations (*n* = 11, 22.9%), and private-sector or technology entities (*n* = 6, 12.5%). The survey examined the perceived significance of digital health competencies within respondents’ organizations, covering technical skills (data analytics, electronic health records, telehealth services, software development and engineering, data protection and privacy, monitoring and evaluation of digital health interventions, health information systems, health technology assessment, and legal/ethical issues in digital health), emerging technologies and digital futures, soft skills (communication and interpersonal skills, problem-solving and critical thinking, ethical and evidence-based decision-making), and project management, leadership, innovation, and entrepreneurship in the health sector.

Overall, 81.3% of respondents rated digital health competencies as highly important for their organization, and 89.6% indicated willingness to employ graduates of the proposed MSc in Digital Health program. A multidisciplinary skill set was identified as essential: core technical competencies included data analytics, electronic health records, and health information systems, while soft skills such as communication, leadership, problem-solving, and ethical decision-making were also emphasized. As summarized in Table 3, the most frequently identified emerging-technology competencies were AI and machine learning (48.0%) and big data and analytics (41.7%), while key gaps in the previous curriculum centered on data analytics, AI, epidemiology, biostatistics, and health information systems.

**Table 3:** Thematic core technical competencies in the reviewed curricula.

Theme	Sub-theme	Frequency (%)
Emerging Technologies	Artificial Intelligence (AI) and Machine Learning (ML)	48.0%
	Big Data and Analytics	41.7%
	Blockchain Technology	18.8%
	Virtual Reality (VR) and Augmented Reality (AR)	12.5%
	Internet of Things (IoT)	10.4%
Data Management and Analytics	Data Science and Predictive Analytics	35.4%
	Health Information Systems (HIS) and Interoperability	29.2%
	Cybersecurity and Data Privacy	25.0%
	Data Visualization	16.7%
Public Health and Clinical Skills	Epidemiology and Biostatistics	20.8%
	Clinical Workflows and EHR Management	18.8%
	Telemedicine and Remote Monitoring	14.6%
Soft Skills and Leadership	Ethical and Legal Issues in Digital Health	22.9%
	Leadership and Change Management	18.8%
	Health Entrepreneurship and Innovation	12.5%
	Project Management	10.4%

### 3.3 Qualitative insights from alumni interviews and open-ended survey responses

Thematic analysis of alumni interviews and open-ended survey responses identified key competencies and structural considerations for the MSc Digital Health program. The findings clustered around five broad areas: advanced computing and emerging technologies; applied health data science and analytics; health systems architecture and interoperability; experiential learning and program delivery; and governance, cybersecurity, leadership, and professional ethics.

Stakeholders emphasized the need to strengthen advanced technical competencies beyond basic information technology skills. Artificial intelligence (AI) and machine learning (ML) were particularly highlighted, including natural language processing, deep learning, computer vision, predictive analytics, and robotics. One stakeholder noted, “*Artificial Intelligence is broad. The curriculum needs to be carefully designed to ensure it covers all the subfields of AI, including natural language processing, machine learning, especially deep learning, computer vision, and robotics and their applications in health*.” (Respondent #14). Respondents also identified emerging technologies such as augmented and virtual reality, mixed reality, cloud computing, blockchain, generative AI, and 3D printing as relevant areas for future digital health practice.

Stakeholders placed strong emphasis on applied health data science and analytics. Respondents recommended practical training in statistical and analytical platforms, including Microsoft Excel, SPSS, R, and Stata, as well as programming, data management, big data analytics, data warehousing, and bioinformatics. One stakeholder emphasized, “*It is key to teach students how to go about analyzing their data using different platforms such as advanced Microsoft Excel, SPSS, R, Stata, etc.*” (Respondent #28). The importance of developing and using locally relevant datasets was also highlighted, with one respondent noting, “Emphasis can be given to creation and use of local datasets for analytics.” (Respondent #9).

The findings further highlighted the importance of health systems integration, interoperability, and foundational health sciences. Stakeholders emphasized interoperability, clinical decision-support systems, digital health usability, and alignment of digital technologies with healthcare workflows and national standards. Foundational competencies in epidemiology, biostatistics, health classification, health economics, and health-system functioning were also considered important. One stakeholder suggested, “*In order to align with digital health implementation, I propose additional skills about the healthcare system and the interrelationship of different blocks*.” (Respondent #39). These findings reflect the need for graduates to understand digital health within the broader health-system context rather than as a purely technological field.

Experiential learning and flexible program delivery were also prominent (35). Stakeholders recommended real-world projects, internships, practicums, and hospital attachments to strengthen the application of classroom knowledge. One stakeholder stated, “*I have an opinion that the graduates in digital health… should be given a year or more to practically work on their skills in hospitals or a given project.*” (Respondent #29). Given the anticipated diversity of entrants from clinical and computer science backgrounds, differentiated learning pathways were proposed to accommodate differences in prior competencies. Flexible delivery options, including evening, hybrid, and online learning, were also recommended to improve accessibility for working professionals. As one stakeholder suggested, “*Due to the rise in technology, I believe the university should be able to offer this course also online with advanced technology in teaching and instruction*.” (Respondent #32).

Finally, stakeholders emphasized governance, cybersecurity, leadership, ethics, and change management as essential components of digital health education. Cybersecurity, data protection, professional ethics, and responsible use of health data were considered critical for protecting patients and strengthening trust in digital health systems. Strategic leadership and transformational skills were similarly emphasized, particularly the ability to bridge technology and healthcare, manage organizational change, and promote patient-centered, equitable, and sustainable digital transformation. One stakeholder emphasized, “*Strategic leadership, using technology tools as building blocks to enable transformation and change… while promoting equity, equality, efficiency and effectiveness*.” (Respondent #46).

Overall, the qualitative findings supported a multidisciplinary approach to digital health education that extends beyond traditional health informatics to encompass advanced technologies, data science, health-system integration, practical implementation, governance, and leadership. These findings informed the development and refinement of the revised MSc Digital Health curriculum.

### 3.4 Stakeholder workshop validation outcomes

The stakeholder workshop reviewed and validated the draft MSc Digital Health curriculum developed through benchmarking, the tracer study, gap analysis, and stakeholder consultations. Participants generally endorsed the proposed curriculum and provided recommendations to strengthen its relevance, practical orientation, and responsiveness to emerging digital health needs. Table 2 summarizes key feedback and the corresponding curriculum actions.

### 3.5 Resulting program structure and multidisciplinary curriculum

To address the multifaceted nature of digital transformation and ensure alignment with complex global health requirements, the MSc in Digital Health at MUHAS adopts a strictly multidisciplinary admissions policy. The program enrolls candidates with bachelor’s degrees from diverse professional backgrounds, fostering a collaborative learning environment that bridges clinical practice and technological innovation. Eligible applicants include graduates from clinical and health-related sciences, such as medicine, dentistry, nursing, pharmacy, laboratory sciences, public health, and environmental health, as well as those from technical and analytical sciences, including computer science, information technology, informatics, engineering, and statistics. The program also admits candidates from social and management sciences such as demography, economics, and business administration.

#### 3.5.1 Curricular framework

The MSc in Digital Health curriculum is structured as a progressive, integrated framework that equips students with foundational, core, and advanced competencies in digital health. The program begins with public health and research foundations, including epidemiology, biostatistics, and the principles of One Health. It further incorporates management and governance components such as health policy, leadership, and legal and ethical issues in digital health. Core digital health competencies include software engineering in health systems, data science, applications of AI and ML, and cybersecurity and data protection. The curriculum also emphasizes implementation science, focusing on the design, evaluation, and adoption of digital health interventions, as well as health entrepreneurship and advanced data analytics. A practicum component and a final dissertation ensure applied learning and research competence. The program further offers specialization tracks in digital health leadership, systems development, and health data science and AI. Figure 1 illustrates the curriculum structure.

**Figure 1:**
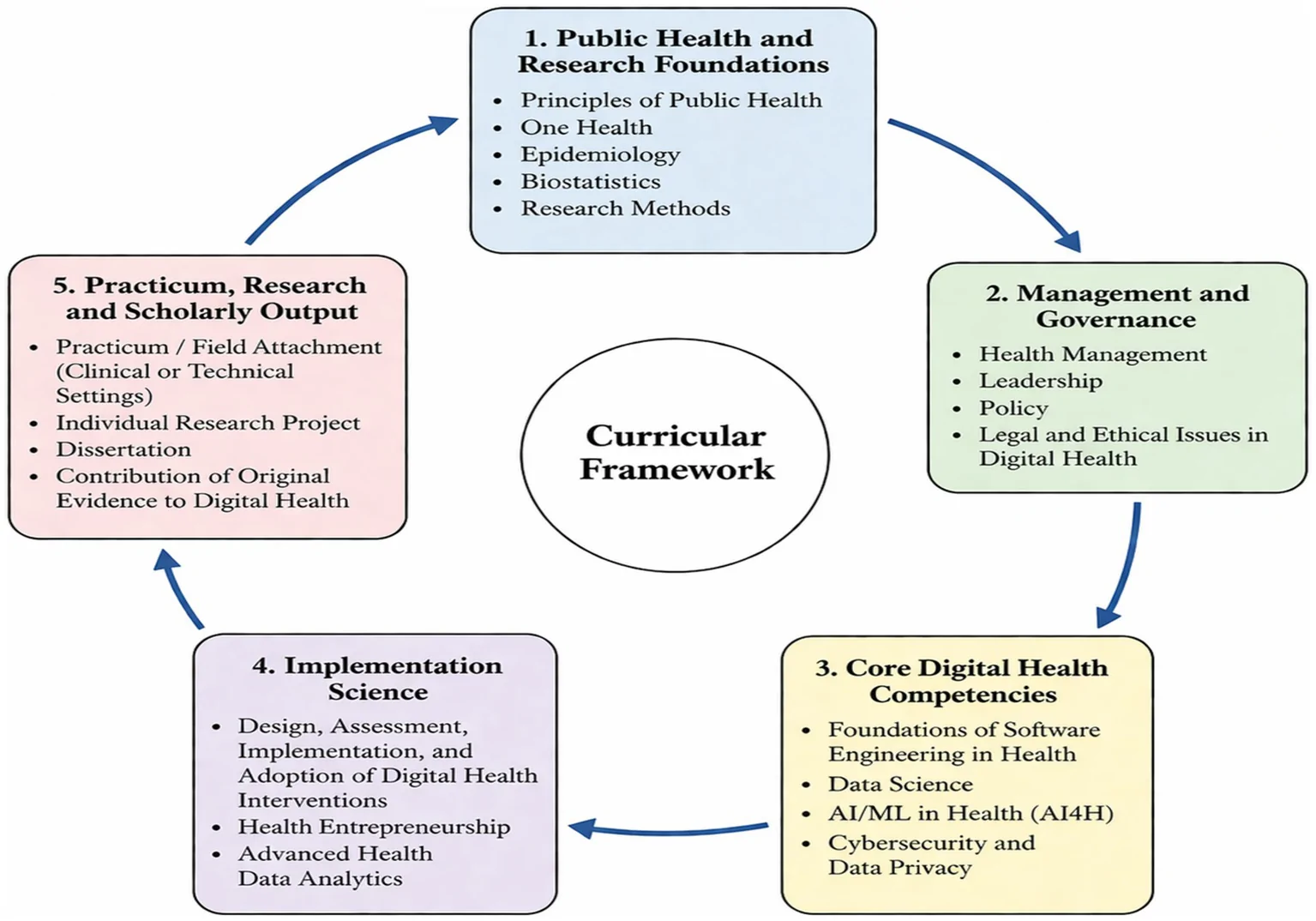
Curriculum framework for the MSc in digital health program at MUHAS.

A critical component of the pedagogy is the requirement for a practicum or field attachment, providing students with hands-on experience in real-world clinical or technical settings. The program culminates in an individual research project and dissertation, allowing students to contribute original evidence to the digital health domain.

#### 3.5.2 Specialization tracks

The program is structured to cultivate graduates specializing in three distinct tracks (Table 4), designed so that MUHAS graduates meet the workforce-capacity indicators identified by the Global Digital Health Index (GDHI), particularly regarding the development of trained professionals to support the country’s digital health needs.

**Table 4:** Specialization tracks of MSc in Digital Health at MUHAS.

Track	Core focus areas
1. Digital Health Leadership	Policy development, strategy, digital and data governance frameworks, and implementation research.
2. Digital Health Systems Development	Software design, system development and deployment, systems integration, and regional interoperability standards.
3. Health Data Science and AI	Machine learning, intelligent systems, big data analytics, and predictive modelling.

## 4 Discussion

The findings of this study reflect a broader global shift in workforce demand from traditional health information management toward a comprehensive digital health paradigm. The redesigned MSc Digital Health curriculum responds to this shift by integrating advanced informatics, data science, and interdisciplinary training, consistent with global recommendations (2, 39).

MSc programs in digital health are evolving rapidly worldwide, aiming to equip graduates with multidisciplinary competencies spanning informatics, data science, leadership, ethics, implementation science, and emerging technologies such as AI, ML, blockchain, and edge computing (9–12, 34). Curricula are often mapped to established competency frameworks, but content, structure, and emphasis vary significantly across institutions and countries (9, 12–15). While core domains such as health informatics, data analytics, digital communication, privacy/security, and leadership are common, gaps remain in cybersecurity, telehealth, ethics, and practical skill development (13, 15), a pattern mirrored in our own benchmarking (Section 3.1) and survey findings (Section 3.2). Interdisciplinary approaches and stakeholder engagement are increasingly recognized as essential for curriculum relevance and workforce readiness (12, 16, 17), yet integration into core curricula remains inconsistent, with many programs offering digital health as electives rather than longitudinal or mandatory tracks (10, 14, 18).

Effective implementation of digital health initiatives relies on coordinated contributions from strategic leaders, clinical champions and frontline staff, information-technology and evaluation teams, and broader stakeholder networks, with competence and engagement in each role—cultivated through training, support, and co-design, repeatedly identified as essential for sustainable impact (19–22). This is consistent with the multi-stakeholder engagement approach adopted in developing the MUHAS curriculum. Recent regional evidence reinforces this point: the WHO Regional Office for Africa’s State of the Health Workforce in Africa 2026 report recommends integrating digital health, AI, and simulation-based learning into competency-based health professions education to strengthen workforce retention, performance, and sustainability (36), and similarly recommends investment in simulation laboratories and digital learning platforms to expand clinical education capacity in underserved settings an approach reflected in our recommendations (Section 6).

The inclusion of AI, big data analytics, and biostatistics competencies is intended to enable future graduates to support data-driven decision-making and improve health system performance (23, 24), while the integration of ethics, governance, and cybersecurity is intended to address growing concerns about data privacy and the responsible use of digital technologies (2). Whether these intentions translate into practice will depend on implementation, and is a question for future evaluation rather than the present study.

In 2025, the WHO Science Council highlighted persistent challenges in harnessing digital technologies in the health sector relative to other industries, warning that inadequate access to high-quality digital health services risks widening health inequities within and between countries, and proposed four action areas: connect, educate, invest, and evaluate. Under “educate,” it recommended standardized digital health competency frameworks, curriculum reform, recognition of digital health qualifications, and clear career pathways (25), reinforcing the need for multisectoral collaboration among ministries of health, education, finance, workforce, and ICT, together with academic institutions and professional bodies, the same coalition of actors engaged in the present curriculum development process.

Several barriers to implementing these recommendations are documented in the literature and are relevant to the MUHAS context. Fragmented funding and limited sustained investment in digital public infrastructure continue to widen the digital divide in LMICs (10, 25); already crowded medical curricula make integrating digital health content a trade-off against core subjects (10); and a global shortage of faculty with digital health expertise limits institutional capacity to deliver high-quality training (2, 4). Infrastructure limitations, particularly constrained access to digital platforms and connectivity, further reduce opportunities for hands-on training (10, 25), while introducing digital technologies without adequate workforce support, fair compensation, and psychosocial support may increase workload and contribute to burnout and attrition (36). The rapidly evolving nature of digital health technologies also makes it difficult to design stable, future-proof curricula, as required skill sets continually change (10), compounded by fragmented health information systems, staff turnover, and limited organizational support (26–30).

A skilled data and technology workforce is essential to supporting public health amid modernization; multiple training options and sustained funding are needed to bridge skills gaps and upskill the existing workforce, with strong academic–practice partnerships identified as a promising strategy (31). Consistent with this, a recent Ukrainian study recommended that medical education institutions systematically map their existing curricula against established competency-framework components to identify gaps (10, 32), an approach directly reflected in the document review and benchmarking exercise reported here (Section 3.1).

Taken together, the benchmarking findings confirm that digital health curricula internationally tend toward institutional specialization rather than harmonization, producing redundancy in foundational competencies alongside underrepresentation of advanced domains such as applied machine learning and AI system evaluation. The MSc Digital Health program at MUHAS was designed to address this gap directly by combining foundational and advanced competencies within a structured, competency-based framework informed by benchmarking, stakeholder survey and interview data, and workshop validation. Its contribution to producing a “future-ready” digital health workforce, however, remains a proposition to be tested once the program is implemented, rather than a finding of the present study (see Section 5).

## 5 Limitations of the study

This study has several limitations that should be considered when interpreting the findings. First, the quantitative survey sample (*n* = 48) is relatively small and was drawn using a purposive sampling approach, which may limit statistical precision and the representativeness of findings relative to the full population of digital health stakeholders in Tanzania. Second, the study was conducted within a single national and institutional context (MUHAS, Tanzania), which may limit the direct transferability of specific findings to other countries or institutions, although the overall process is intended to be adaptable to similar LMIC settings. Third, the purposive selection of survey respondents, alumni interviewees, and workshop participants may have introduced selection bias, potentially over-representing stakeholders already engaged with MUHAS or with digital health initiatives and under-representing other perspectives, such as rural health facilities or smaller private-sector actors. Fourth, this study describes the curriculum development process and does not report on educational or workforce outcomes; no data are yet available on TCU accreditation status, student enrolment, graduate competencies, employment outcomes, or the program’s contribution to national digital health initiatives, as the program has not yet produced graduates. Fifth, the benchmarking exercise relied on publicly available curriculum documents, which may not fully capture how programs are actually delivered in practice. Finally, alumni interviews relied on retrospective self-report, which may be subject to recall bias, and the study did not include a formal quantitative mapping of the curriculum against GDHI maturity-model indicators. Future evaluation studies will need to assess the effectiveness and impact of the MSc in Digital Health program, including graduate competencies, employment outcomes, employer satisfaction, and contributions to national digital health initiatives.

## 6 Conclusion

This study documents a strategic, evidence-based process for developing the MSc Digital Health program at MUHAS, responding to a documented gap in digital health competencies among the health workforce in Tanzania and similar LMIC settings. By combining a structured gap analysis and benchmarking exercise, quantitative and qualitative stakeholder engagement, and expert workshop validation, the resulting curriculum integrates emerging technologies, practical training, and interdisciplinary competencies. It is intended to position graduates to contribute to digital health transformation once the program is implemented.

Developing the curriculum required engagement from multi-sectoral stakeholders, including ministries responsible for health, education, workforce, and the ICT/digital economy, as well as accreditation authorities and professional associations in health, informatics, and digital health. Continued engagement of this kind will be important for developing recognized career paths and regulatory frameworks for digital health professionals, to make full use of digital technology in bridging workforce shortages. Establishing professional trajectories that formally recognize the distinct contributions of digital health specialists, including informatics specialists, software engineers, data scientists, AI engineers, AI ethicists, data protection officers, and cybersecurity experts, together with regulation promoting the ethical, responsible, and equitable use of digital and data technologies, will be important complements to curriculum reform of the kind described here.

## 7 Recommendations

A standardized national digital health competency framework should be established to clearly define core, intermediate, and advanced competencies required for the digital health workforce, addressing the fragmentation across curricula identified in the benchmarking exercise (Section 3.1). This framework should be aligned with international standards and guidelines from the WHO and the International Medical Informatics Association (IMIA) to ensure consistency, quality, and global relevance.

Digital health competencies should be mainstreamed across all health training programs by integrating them into both undergraduate and postgraduate curricula. This approach ensures that all health professionals, regardless of specialization, are equipped with essential digital skills, rather than limiting such competencies to specialized programs.

Collaboration among academia, industry, and government should be strengthened to ensure training remains responsive to evolving workforce demands, consistent with stakeholder feedback reported in Section 3.3. Formal partnerships should be established to support internships, co-development of curricula, and opportunities for students to engage in real-world digital health problem-solving.

Governments and academic institutions should invest in digital health training infrastructure to support effective learning. This includes establishing simulation laboratories and digital innovation hubs, and providing access to real-world datasets and advanced analytical tools to enhance practical skills development.

Emerging technologies such as AI, ML, blockchain, cybersecurity, and big data analytics should be embedded as core components of digital health curricula, reflecting the high priority these areas received in both the benchmarking exercise and the stakeholder survey (Table 3). Treating these areas as central rather than optional ensures that graduates are adequately prepared for the rapidly evolving digital health landscape.

Faculty capacity in digital health must be strengthened through continuous professional development initiatives. These programs should build expertise in data science, health informatics, AI, and digital health governance to support effective teaching and mentorship.

Finally, all academic training programs should align with national and global digital health strategies, including the Tanzania Digital Health Transformation Strategy (2025–2030), to ensure training remains relevant, sustainable, and capable of supporting ongoing digital transformation in the health sector. The MSc Digital Health program should be prospectively evaluated, for example, by assessing graduate competencies, employment outcomes, employer satisfaction, and contributions to digital health initiatives, to determine whether it achieves its intended workforce impact.

## Data Availability

The original contributions presented in the study are included in the article/supplementary material, further inquiries can be directed to the corresponding author.
